# Long Term Effects of Tear Gases on Respiratory System: Analysis of 93 Cases

**DOI:** 10.1155/2014/963638

**Published:** 2014-07-22

**Authors:** Peri Arbak, İlknur Başer, Özlem Ozdemir Kumbasar, Füsun Ülger, Zeki Kılıçaslan, Fatma Evyapan

**Affiliations:** ^1^Department of Chest Diseases, Duzce University Medical Faculty, Düzce, Turkey; ^2^Emergency Department, Ankara Numune State Hospital, Ankara, Turkey; ^3^Department of Chest Diseases, Ankara University Medical Faculty, Ankara, Turkey; ^4^Department of Chest Disease, Private Practice, Ankara, Turkey; ^5^Department of Chest Diseases, Istanbul University Medical Faculty, Istanbul, Turkey; ^6^Department of Chest Diseases, Pamukkale University Medical Faculty, Denizli, Turkey

## Abstract

*Aim*. This study aimed to assess the long-term respiratory effects of tear gases among the subjects with history of frequent exposure.* Materials and Methods*. A questionnaire by NIOSH and pulmonary function tests was performed in 93 males exposed to the tear gases frequently and 55 nonexposed subjects.* Results*. The mean numbers of total exposure and last 2 years exposure were 8.4 ± 6.4 times, 5.6 ± 5.8 times, respectively. Tear gas exposed subjects were presented with a higher rate for cough and phlegm more than 3 months (24.7% versus 11.3%, *P* > 0.05). Mean FEV1/FVC and % predicted MMFR in smoker exposed subjects are significantly lower than those in smoker controls (81.7% versus 84.1%, *P* = 0.046 and 89.9% versus 109.6%, *P* = 0.0004, resp.). % predicted MMFR in nonsmoker exposed subjects is significantly lower than that in nonsmoker controls (99.4% versus 113.1%, *P* = 0.05). Odds ratios for chest tightness, exercise dyspnea, dyspnea on level ground, winter morning cough, phlegm, and daily phlegm were increased almost 2 to 2.5 folds among tear gas exposed subjects.* Conclusion*. The rates for respiratory complaints were high in the case of the exposure to the tear gases previously. Tears gas exposed subjects were found to be under the risk for chronic bronchitis.

## 1. Introduction

Tear gases have been used as “Riot Control Agents” for a long time by police forces. The mostly used agents among tear gases are 2-chlorobenzylidene malononitrile (CS), oleoresincapsicum (OC), and chloroacetophenone (CN) [1, 2]. The usage types of tear gases are gas bomb and spray. The main effect of “Riot Control Agents” is irritation of mucosal surfaces.

Respiratory symptoms related to CS are nasal irritation, rhinorrhea, cough, and breathlessness. Although the respiratory symptoms are transient in nature, laryngospasm, pulmonary edema, and reactive airways dysfunction have also been reported [3, 4]. Exposure in closed spaces increases the detrimental effects of CS. Pulmonary function deterioration and respiratory complaints might be observed several months after the cessation of exposure to CS [1]. Karagama et al. reported that out of 34 persons exposed to CS in a closed area 23 had respiratory complaints after one hour after the cessation of exposure. They followed the cases and showed that respiratory symptoms persisted for 10 months in 5 subjects [5].

Other common tear gas, OC, may also cause sore throat, cough, wheezing, shortness in breath, laryngospasm, and rarely respiratory arrest [6]. Steffee et al. investigated custody-deaths related to pepper spray. In that report, the victim was an asthmatic who had been sprayed 10 to 15 times with pepper spray. Postmortem examination revealed severe epithelial lung damage, and the cause of death was severe acute bronchospasm, probably precipitated by the use of pepper spray.

In the last few years, the inappropriate use of “Riot Control Agents” has been a public concern in Turkey. Turkish Police Department declared that they mostly used OC and CS as tear gases for controlling protest actions. Some tear gas related deaths have been reported. Turkish Medical Association attempted to forbid the use of tear gases since those turned out to be a huge public health problem [7].

Turkish Thoracic Society has faced tremendous amount of questions about tear gases since last years. The aim of this study was to compare the respiratory complaints and pulmonary functions of 93 subjects who were frequently exposed to tear gases and 55 nonexposed subjects as a control group.

## 2. Materials and Methods

This study was conducted between March 2012 and October 2012. White collar workers, teachers, students, journalists, professional unionists, and political activists with frequent exposure were enrolled into the study group (total 93 subjects). All were men and were exposed to the tear gases much more frequently compared to general population. Fifty-five nonexposed subjects—health care workers, bureau workers, and faculty members—matched for age, gender, and smoking status formed the control group. We used a questionnaire about exposure (the total number of exposure times for last 2 years, the type of tear gases, and distance from the source), hospitalization after the exposure, and self-prevention measures against exposure. We also used an additional questionnaire, Initial Questionnaire of the NIOSH-Occupational Asthma Identification Project. We performed pulmonary function tests with same equipment (Vitalograph Alpha) and the same subject (PA) according to ATS criteria [8]. Spirometry measurements—forced vital capacity (FVC) and forced expiratory volume in 1 s (FEV1) and maximal midexpiratory flow rate (MMFR)—are used as percent of predicted values.

Spirometric measurements and questionnaires were done in the centre of United Metalworkers' Union, İstanbul, the center of Confederation of Public Workers' Unions, Ankara, and Duzce University, Medical Faculty, Department of Pulmonary Disease, Duzce.

An ethical approvement was obtained from Ethical Committee of Istanbul University.

SPSS-13.0 statistical programme was used. The Chi-square test was used for testing differences in the prevalence of respiratory symptoms, other symptoms, smoking status, and asthma history among the groups. Odds ratios for the presence of respiratory symptoms were calculated in the case of the exposure to tear gases. Comparison of spirometric measurements was performed by *t*-test for two independent samples. A *P* value less than 0.05 was considered as statistically significant.

## 3. Results

Demographic and health aspects of exposed subjects and controls are shown in Table 1.

The mean number of total exposure times (life-long exposure) to tear gases was 8.4 ± 6.4 times (min: 1, max: 30) and the mean number of the last 2 years exposure times was 5.6 ± 5.8 times (min: 1, max: 40).

The mean daily cigarette consumption among exposed subjects (22.5 ± 12.1) was significantly higher than that in controls (15.2 ± 7.7, *P* = 0.003).

The mean beginning age (years) of smoking among exposed subjects (18.5 ± 4.8) was not different than that in controls (18.7 ± 5.0, *P* > 0.05).

Respiratory complaints of subjects are shown in Table 2.

Exposed subjects have had higher respiratory complaints rates except wheezing and cough for 3 months than controls.

**Figure 1:**
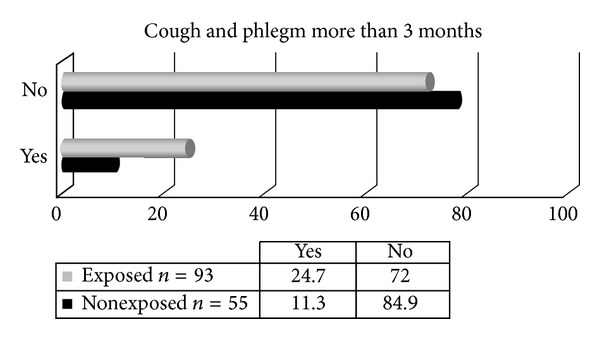
Cough and phlegm more than 3 months among subjects.

The incidence of both cough and phlegm complaints more than 3 months is seen in Figure 1.

Tear gas exposed subjects were presented with a higher rate for cough and phlegm of more than 3 months (*P* > 0.05).

**Figure 2:**
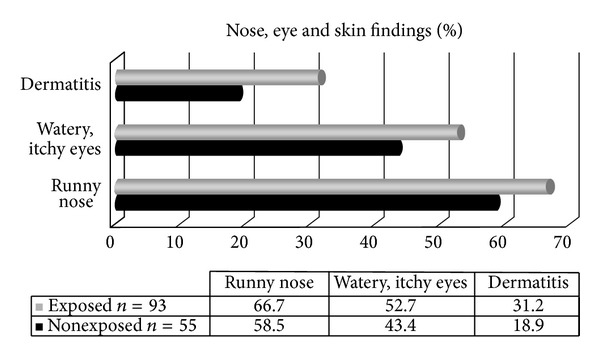
Nose, eye, and skin complaints among tear gas exposers.

The rates for runny nose, watery eyes, and dermatitis during the last 12 months are shown in Figure 2.

Higher rates for runny nose, watery eyes, and dermatitis were seen among tear gas exposed subjects compared to nonexposed ones but the differences were not statistically significant.

Pulmonary function data of subjects are shown in Table 3.

Mean FEV1/FVC and % predicted MMFR in smoker exposed subjects are significantly lower than those in smoker controls (*P* = 0.046 and *P* = 0.0004, resp.). % predicted MMFR in nonsmoker exposed subjects is significantly lower than that in nonsmoker controls (*P* = 0.05).

Odds ratios of respiratory complaints for tear gas exposed group are shown in Table 4.

## 4. Discussion

Current study showed marked increase in respiratory complaints including cough, phlegm, dyspnea, and chest tightness among subjects frequently exposed to tear gases compared to nonexposed subjects since last year. A significant decrease in the mean % predicted maximal expiratory flow rate in tear gas exposed subjects was detected and both of the mean % predicted maximal expiratory flow rate and FEV1/FVC ratio significantly decreased among cigarette smokers.

Previous studies have focused on the acute effects of tear gases and most of them are based on experimental studies of voluntary subjects in the laboratory basis. In the case of the exposure to OC, transient respiratory complaints that ranged from coughing and shortness of breath to gasping for breath were linked to the inflammation and irritation. Authors also reported excess nasal and tracheobronchial secretions, sneezing, and changes in breathing pattern [9–11]. Studies focused on the chronic effects of OC are scarce and most of them are associated with occupational exposure to capsaicinoids. These studies typically reported initial respiratory symptoms similar to those seen with acute exposure (e.g., cough, runny nose, and sneezing). As seen in byssinosis these symptoms decreased and often were completely reversed, with continued exposure. In a study including 22 hot pepper workers with chronic exposure to hot chili powder showed chronic cough in 59% of the exposed workers, compared to 21% of the controls. The exposed workers also had more common complaints of chest discomfort, shortness of breath, and stuffy or runny nose [12]. Another study showed increased respiratory symptoms (upper respiratory tract irritation, sneezing, and runny nose) in 49% of male spice grinders related to workplace exposure [13]. Morning cough (32%) and daily cough during winter (39%) were high; furthermore morning phlegm (28.0%), daily phlegm in winter (42.0%), and phlegm more than 3 months (26%) were accompanying to cough among tear gas exposed subjects in the present study. It has been postulated that the lesser exposure to capsaicinoids during protest actions than that in spice processing and exposure to tear gases at the outdoors led to a lower chronic cough rate. Tear gas exposed subjects also presented high rates of dyspnea (44%), chest tightness (37.6%), and exercise dyspnea in the last year (43%). Comparably to the spice workers, OC exposed subjects have had high rates for irritation symptoms such as runny nose (66.7%), watery eyes (52.7%), and dermatitis (31.2%). The results of tear gas exposed subjects were similar to those in two studies that evaluated the long term respiratory effects in the case of the exposure to sulfur mustard [14]. A study of 197 Iranian veterans, 10 years after a single heavy exposure to sulfur mustard, revealed a series of delayed destructive pulmonary sequela such as chronic bronchitis (58%), asthma (10%), bronchiectasis (8%), large airway narrowing (9%), and pulmonary fibrosis (12%) [14]. Sixteen to twenty years after exposure, main respiratory complications were diagnosed as chronic obstructive pulmonary disease (35%), bronchiectasis (32.5%), asthma (25%), large airway narrowing (15%), pulmonary fibrosis (7.5%), and simple chronic bronchitis (5%) [15]. Cough and phlegm more than 3 months incidence was 24.7% in the present study. In a review by Hu et al., in the case of pure CS exposure, shopkeepers and their families in communities nearby where the demonstrations took place complained of cough and shortness of breath that persisted for several weeks after political demonstrations in Seoul, July 1989 [1]. Coughing, wheezing, and dyspnea persisted for two years after short term exposure to CS in a previously well 21-year-old woman [4]. Among 34 young adults exposed to CS in a coach, 5 of them had respiratory symptoms, 2 had worsening of asthma, 2 had decreased exercise tolerance, and 1 complained of coughing fits after exercise at the examination done 8 to 10 months after the exposure [5]. Respiratory complaint rates between 26% and 44% among subjects exposed to tear gases in a regular basis (mean life-long exposure number 8.4 and the mean last 2 years exposure number 5.6) were compatible with the knowledge on the long term effects of CS in the literature.

There is little knowledge on the long term effects of capsaicinoids on pulmonary functions and most of them are based on occupational exposure. A study of 22 hot pepper workers chronically exposed to hot chili powder and 19 unexposed workers in the same plant showed no significant decrease in baseline pulmonary function in exposed group. History of asthma did not affect pulmonary function tests [12]. In another study, Lankatilake and Uragoda evaluated pulmonary function in 25 male workers of chili grinding factories in Sri Lanka with an average of 6.6 years of exposure. Pulmonary function measurements in the exposed workers were not different from the controls, and there was no difference between the pre- and postshift pulmonary function measurements in exposed workers after a weekend of no exposure [16]. The mean FEV1/FVC ratio of tear gas exposed and smoker subjects was lower than that of exposed ones in the present study. Furthermore significant decreases in mean % predicted MMFRs of both smoker and nonsmoker tear gas exposed subjects showed an obstructive pattern in pulmonary function. The additive mixtures used in OC and CS, exposure to both OC and CS during protest actions, and massive use of tear gases may be responsible for marked decrease in MMFR among tear gas exposed subjects. Present study is the unique one for reflecting the long term respiratory health consequences of subjects faced with tear gases in real life such as protest actions. Limited reliability of the knowledge on the exposure to tear gases is one of the limitations of the present study. Lack of the memory when answering the questionnaire by NIOSH is the second one. At least two types of tear gases (OC and CS) and their additives effects on the respiratory system have been evaluated and none of them have not been incriminated for the obstructive pattern alone.

In the light of the results in the present study, consider the following.

- Being exposed to tear gases decreases maximal mid expiratory flows in both smokers and nonsmokers.
- Being both smoker and exposed to tear gases decreases FEV1/FVC ratio.
- Chronic bronchitis is increased among exposed subjects although this rate does not reach statistical significance.
- Almost all of the complaints related to the upper and lower respiratory tract, eyes, and skin increase in the case of the exposure to the tear gases.

Additional efforts are necessary for searching the hazardous effects of tear gases to be able to forbid the use of tear gases as for controlling the protest actions.

## Figures and Tables

**Table 1:** Demographic and health aspects of exposed subjects and controls.

	Exposed subjects *n* = 93	Controls *n* = 55	*P*
Age (years)	38.9 ± 9.3	36.3 ± 8.6	>0.05
Height (cm)	175.4 ± 7.4	175.9 ± 6.3	>0.05
Weight (kg)	81.3 ± 14.3	81.4 ± 12.2	>0.05
Smokers	70 (75.3%)	35 (66.0%)	>0.05
Subjects with asthma history	7 (7.5%)	5 (9.4%)	>0.05

**Table 2:** Respiratory complaints of subjects.

	Exposed subjects *n* = 93	Controls *n* = 55	*P*
Wheezing	56 (60.2%)	24 (45.3%)	>0.05
Dyspnea last year	41 (44.1%)	15 (28.3%)	0.043
Chest tightness last year	35 (37.6%)	8 (15.2%)	0.003
Exercise dyspnea last year	40 (43.0%)	12 (22.6%)	0.010
Dyspnea on level ground	59 (63.4%)	17 (32.1%)	0.0002
Winter morning cough	30 (32.3%)	7 (13.2%)	0.008
Winter daily cough	36 (38.7%)	12 (22.6%)	0.034
Cough for 3 months	25 (26.9%)	8 (15.1%)	>0.05
Winter morning phlegm	26 (28.0%)	6 (11.3%)	0.014
Winter daily phlegm	39 (41.9%)	11 (20.8%)	0.007
Phlegm for 3 months	24 (25.8%)	6 (11.3%)	0.028

**Table 3:** Pulmonary functions of subjects.

% predicted	Exposed subjects *n* = 93 (smokers = 70)	Controls *n* = 55 (smokers = 35)	*P*
FVC	97.3 ± 11.9	89.9 ± 11.7	0.0003
Smokers FVC	97.3 ± 11.9	91.2 ± 12.2	0.018
Nonsmokers FVC	97.4 ± 12.0	87.8 ± 11.1	0.013
FEV1	96.9 ± 12.9	95.9 ± 12.0	>0.05
Smokers FEV1	96.0 ± 13.1	96.8 ± 13.7	>0.05
Nonsmokers FEV1	99.5 ± 12.1	94.6 ± 8.9	>0.05
FEV1/FVC	82.4 ± 6.9	84.2 ± 4.7	>0.05
Smokers	81.7 ± 7.4	84.1 ± 4.6	0.046
Nonsmokers	84.3 ± 5.1	84.4 ± 5.3	>0.05
MMFR	92.3 ± 25.1	110.4 ± 24.5	<0.001
Smokers MMFR	89.9 ± 25.9	109.6 ± 26.8	0.0004
Nonsmokers MMFR	99.4 ± 21.5	113.1 ± 21.3	0.05

**Table 4:** Odds ratios of respiratory complaints in tear gas exposed group.

Parameter	Odds ratio	95% confidence interval	
Chest tightness last year	2.493	1.251	4.971
Exercise dyspnea last year	1.900	1.096	3.292
Dyspnea on level ground	1.978	1.298	3.013
Winter morning cough	2.442	1.153	5.172
Winter morning phlegm	2.470	1.086	5.613
Winter daily phlegm	2.021	1.134	3.601
